# Cost of Horse *v.* Motor Traction

**Published:** 1910-02-12

**Authors:** Walter Munn

**Affiliations:** Secretary


### COST OF HORSE *v.* MOTOR TRACTION.

*To the Editor of* THE HOSPITAL.

DEAR SIR,—Practically every doctor who is not already a motorist is seriously interested in the cost of running a motor-car as compared with horses. It is, however, difficult for him to obtain any accurate data on the subject. I send you herewith an estimate for the two, showing capital outlay and annual expenditure. Your readers, particularly those who are considering the matter, would, I think, be interested in such comparison, and some might furnish tables of their own expenses for comparison.

COMPARISON OF ESTIMATED COSTS FOR MEDICAL MEN OF HORSE AND MOTOR TRACTION.
Distance, 5,000 miles a year.

| HORSE TRACTION. *Capital Outlay.* | £ | s. | d. | MOTOR TRACTION. *Capital Outlay.* | £ | s. | d. |
|---|---|---|---|---|---|---|---|
| 2 horses at £50 | 100 | 0 | 0 | 8-h.p. chassis with body, hood, screen, lamps, tools, etc., £275 to £325, say ... | 300 | 0 | 0 |
| 2 sets harness ... | 20 | 0 | 0 | | | | |
| 1 brougham ... | 120 | 0 | 0 | | | | |
| | £240 | 0 | 0 | | | | |

| *Annual Expenses.* | £ | s. | d. | *Annual Expenses.* | £ | s. | d. |
|---|---|---|---|---|---|---|---|
| Coachman ... | 65 | 0 | 0 | Motor man ... | 65 | 0 | 0 |
| Livery ... ... | 6 | 0 | 0 | Livery ... ... | 6 | 0 | 0 |
| Forage and litter for 2 horses ... | 78 | 0 | 0 | Petrol, 200 gallons at 1s. 2d. | 11 | 13 | 4 |
| Shoeing and vet. | 12 | 0 | 0 | Oil and grease... | 2 | 10 | 0 |
| Repairs and tyres | 10 | 0 | 0 | Carriage repairs, etc. ... ... | 10 | 0 | 0 |
| License, carriage | 2 | 2 | 0 | Machinery repairs and replacements ... | 15 | 0 | 0 |
| | | | | Tyres, do. do.... | 20 | 0 | 0 |
| | | | | License, carriage | 2 | 2 | 0 |
| | | | | Do., 2 driving... | 0 | 10 | 0 |
| | £173 | 2 | 0 | | £132 | 15 | 4 |

The estimate for a De Dion Bouton car is considerably higher than the average of the thirty users whose expenses are given in our booklet "The Cost of Motoring."

I have taken 5,000 as the annual mileage, as I believe this is the minimum travelled by a doctor who has a good practice in the suburbs or country, though those who use horses must find it difficult to cover such a distance unless they are almost continually on the road.

The first cost and running expenses for horses can be checked by the experience of many doctors. Those for an 8-h.p. De Dion Bouton car are considerably higher than the expenses of many medical users of De Dion cars, including the writers of the letters quoted in "The Cost of Motoring" enclosed.

The annual expenses will vary with the car and the user. The best car is the cheapest to maintain, and depreciates least in value; but no car will prove economical or satisfactory if it be neglected and not properly lubricated.

Yours truly,
DE DION BOUTON (1907), LTD.,
WALTER MUNN (Secretary).

